# Older Adults' Satisfaction and Compliance of Smartwatches Providing Ecological Momentary

**DOI:** 10.1093/geroni/igaa057.2898

**Published:** 2020-12-16

**Authors:** Charlotte Laborde, Erta Cenko, Mamoun Mardini, Sanjay Ranka, Parisa Rashidi, Todd Manini

**Affiliations:** 1 Institute on aging, gainesville, Florida, United States; 2 University of Florida, Gainesville, Florida, United States; 3 The University of Florida, Gainesville, Florida, United States

## Abstract

The objective of this study was to evaluate satisfaction, usability and compliance using the Real-Time, Online Assessment and Mobility Monitor (ROAMM) application designed for smartwatches. Twenty-eight older adults with knee osteoarthritis (66-88 years old) wore a Samsung Gear S3 smartwatch with ROAMM. While wearing the watch for 13.16 (±2.94) days, participants gave ecological momentary assessments (EMA) of pain intensity, mood and fatigue at random times in the morning, afternoon and evening. EMA compliance rate was 82.5%. Satisfaction ratings were high with 73% reporting being satisfied with the function, 78% reported being satisfied with the comfort and 73% reported as likely to use ROAMM on a daily basis for a one-year research study. Participants reported to reduce watch size and weight, style and increase battery life. Older adults were compliant to ecological assessment of their symptoms and reported positive usability and satisfaction of wearing a smartwatch and ROAMM app.

